# Exploring Variability in Compound Tensification in Seoul Korean

**DOI:** 10.1177/00238309221095479

**Published:** 2022-06-03

**Authors:** Hae-Sung Jeon

**Affiliations:** School of Humanities, Language and Global Studies, University of Central Lancashire, UK

**Keywords:** Korean, compound, gemination, tensification, frequency

## Abstract

In Korean noun-noun compounds, the lenis onset consonant in the second noun is often realized as a tense consonant. Although extensive work has been carried out to clarify its causes and relevant phonological processes, this tensification is deemed not entirely predictable. This paper presents a speech production experiment that confirms the existing findings that the variability in tensification is predictable to a certain extent. The experimental results also showed that the relationship between the predictors and the variability is not linear and that tensification mirrors the cognitively determined boundary strength. Native Korean speakers calibrate the boundary strength by incorporating complex information, such as the word’s length, segment type, frequency, and plausibility of the compound. While a “tight” boundary led to high tensification probability, it was not affected by speaking-rate variation. Furthermore, the perceived compound’s plausibility directly affected the duration of the tensified consonant. Importantly, the findings suggest that speakers’ calibration of the boundary strength is fluid and changeable over time and it affects both phonological and phonetic outputs. Finally, variability in data was reduced for the experimental conditions leading to either extremely high or low tensification probability, and there seemed to be lexicalized exceptions to the general trends.

## 1 Introduction

In Korean compound nouns that consist of two words, W1 and W2, in which the onset of W2 is a lenis consonant, the W2 lenis onset is often tensified, involving a phonemic categorical change from a lenis to a tense consonant (e.g., /nɑlu/ “dock” + /pɛ/ “boat” → /nɑlup*ɛ/ “ferryboat”).^1^ This tensification is known as *sais-soli* /sɑis*oli/ “between-sound” in Korean; it will be referred to here as “compound tensification.” Although literature in English is scarce, compound tensification has been extensively studied by scholars in Korean linguistics for decades, primarily using approaches in formal morphology or phonology. However, compound tensification is highly variable and there has been no satisfactory account of how it is triggered.

The broad aim of the current study was to establish a clearer empirical picture of how scale factors, such as speaking-rate variation, word frequency, and the plausibility of the compound, affect tensification in tandem with some of the factors identified in previous research when the phonological context is controlled. More specifically, the twofold aims were (1) to examine variation in the categorical change from a lenis to tense realization across speakers and compounds in the experimental setting and (2) to investigate consonantal constriction duration as a possible phonetic correlate of the predictors. What has emerged from previous debates is that tensification has a boundary-marking function, and speakers’ phonological decisions are not solely determined by phonological or morphological factors. However, little research using actual speech data has been carried out so far.

Other languages have similar phenomena exhibiting a high degree of between-speaker and-item variability in compound formation. For instance, *rendaku* in Japanese refers to voicing of the W2 onset (e.g., /hon/ “book” voiced in /bunko-bon/ “paperback book” but not in /huru-hon/ “second-hand book”) which is not entirely predictable (see Itô & Mester, 1999, 2003; Kubozono, 2005). The variability in compound stress in English is well-known (e.g., Dressler, 2007; Kunter, 2011). Compounding is an extremely productive process, and such variability suggests a complex relationship between word formation, the architecture of the lexicon, and phonetic outputs (Bürki, 2018). In this sense, carrying out psycholinguistic (e.g., Pollatsek & Hyönä, 2005; Zwitserlood, 1994) and neuroscientific research (e.g., Kobayashi et al., 2014) on compound tensification can shed light in examining the relationship between cognitive process and speech production, but the basis for such investigation is lacking.

The present study aims to offer a useful basis for further empirical studies. In this exploratory study, the factors in the regression analysis were a subset of previously identified factors, the word’s etymological class, length, the presence of an aspirated or tense consonant, and frequency, and additional factors which have not been studied, such as the type of the W2 onset, speaking rate, and native speakers’ rating of the plausibility of the compound. In addition, the present work aimed to go beyond previous literature to examine the duration of the W2 onset closure, as a phonetic property. Duration has been considered a phonetic correlate of scale properties of words, such as their frequency (Pierrehumbert, 2001, 2002, 2006) and probability (e.g., Bell et al., 2020). It was hypothesized that the factors associated with high tensification probability would increase the consonantal closure duration (see Section 2.1). In what follows, consonantal categories in Korean are reviewed first (Section 1.1), followed by a discussion of the factors affecting the occurrence of compound tensification and relevant hypotheses (Section 1.2).

### 1.1 Korean consonants

Korean has three categories of lenis /p, t, k, tɕ/, tense /p*, t*, k*, tɕ*/, and aspirated /pʰ, tʰ, kʰ, tɕʰ/ stops and affricates, and two categories of denti-alveolar fricatives, plain /s/ and tense /s*/. They are all voiceless in accentual phrase (AP) initial position. The AP is a word-sized prosodic unit mainly demarcated by pitch contour (see Jeon, 2015 for a review, and also S.-A. Jun, 1996, 1998, 2005). The consonantal categories are distinguished by a constellation of cues, such as the closure duration, voice onset time (VOT), and voice quality, and F0 of the following vowel. Lenis stops can be slightly aspirated and the following vowel tends to be breathy (Cho et al., 2002). Unlike aspirated or tense stops, AP-medial lenis stops may be voiced intervocalically, and the voicing occurs gradiently (Cho et al., 2002; S.-A. Jun, 1994, 1996; Lisker & Abramson, 1964). The aspirated stops have the longest VOT and the tense stops have the shortest. The vowel following a tense stop shows creaky voice until its mid-point (e.g., Cho & Keating, 2001). AP-initially, F0 in the vowel following a lenis stop tends to be low, but an aspirated or tense stop triggers high F0, while the difference in F0 between the consonant categories is neutralized or reduced AP-medially (see S.-A. Jun, 1996, 1998). These properties are mirrored in the three categories of alveo-palatal affricates (see Shin et al., 2013, sect. 4.2.3).

The acoustic properties of the plain /s/ are associated with either lenis or aspirated stops, in that it can be voiced between vowels in the AP and aspirated AP-initially (see C. B. Chang, 2013; Cho et al., 2002). The vowel onset following the tense /s*/ shows creaky voice, as in the vowel following tense stops and affricates. The frication portion of tense /s*/ has a higher spectral centroid frequency and longer frication duration compared to the plain /s/ in word-initial position (Cho et al., 2002; Yoon, 1999, 2005). The acoustic properties of the lenis and tense consonants relevant to the present study are summarized in Table 1.

**Table 1.** Acoustic Properties of Lenis and Tense Consonants.

Property	Lenis	Tense
Closure duration	Short	Long
Voicing (AP-medial)	Can be voiced	Voiceless
VOT (AP-initial)	Long	Short
F0 at vowel onset (AP-initial)	Low	High
Voice quality	Breathy	Pressed

VOT: voice onset time.

### 1.2 Compound tensification in Korean

In Korean, a lenis consonant is realized as a tense consonant in a range of contexts. For example, tensification occurs following an obstruent (post-obstruent tensing, e.g., /pɑp/ “rice” + /totuk/ “thief”, /pɑptotuk/→/pɑpt*otuk/, “a delicious side dish, lit. rice-thief”), a coronal lenis consonant following /l/ may be tensified (e.g., /kjʌltɑn/→/kjʌlt*ɑn/, “decision”), and a lenis consonant in the verbal suffix can be tensified (e.g., /kɑm-/ “to coil” + /-ko/ connective, /kɑmko/→/kɑmk*o/ “to coil and”). Compounding is often referred to as one of the conditions triggering tensification (see Shin et al., 2013, Chs. 8 and 9 for a summary; also see Zuraw, 2011). For instance, a monomorphemic word (e.g., /tɕɑmtɕɑli/ “dragonfly”) can be disambiguated from a compound with the same underlying phonemic representation, because only the latter undergoes tensification (e.g., /tɕɑm/ “sleep” + /tɕɑli/ “place”, /tɕɑmtɕɑli/→/tɕɑmtɕ*ɑli/ “bed-space”).

There have been extensive debates on the conditioning factors and relevant phonological rules for compound tensification. For instance, morphological and semantic triggers, such as a subordinate relation between W1 and W2 and animacy, have been proposed (see Chung, 1980, Ch. 3 and Im, 1981 for a thorough review); for instance, /pi/ “rain” is tensified in /pom + p*i/ “spring(‘s) rain” but not in /nun + pi/ “snow and rain,” and /tɑli/ “leg” is tensified in /tɕʰɛksɑŋ + t*ɑli/ “a desk’s leg” but not in /sɑlɑm + tɑli/ “a person’s leg.” It has been suggested that tensification is the outcome of abstract phonological rule application, such as /t/-insertion (C.-W. Kim, 1970; Kim-Renaud, 1974), [Ɂ]-insertion (Martin, 1954; Oh, 1987), phonological C slot insertion (W.-H. Chang, 2004), or gemination of the W2 onset consonant (Cook, 1987), to cite just a few (see J. Jun, 2018 for a recent survey). For instance, the /t/-insertion account suggests that /t/ is inserted at the word boundary, for example, /nɑlu/ “dock” + /t/ + /pɛ/ “boat”, /nɑlutpɛ/, then the Post-obstruent tensing rule is applied, /nɑlutpɛ/→/nɑlutp*ɛ/, and then the output undergoes consonant cluster simplification, producing the final output /nɑlup*ɛ/ “ferryboat.” Some researchers have suggested that compound tensification indicates the presence of an intermediate-strength boundary above the morpheme level and tight joining of the constituents (Ahn, 1985; Chung, 1980; Oh, 1987).

Korean linguists have expressed the intuition that there are some systematic triggers. However, none of the proposals has provided a satisfactory explanation for when tensification occurs, and the application of any rules results in too many exceptions. The traditional analysis is also limited in that it was mainly based on individual researchers’ intuitions, while studies involving multiple participants report between- and within-speaker variation (National Institute of the Korean Language [NIKL], 2016, p. 35, also see Ito, 2014; S. Kim, 2017; Zuraw, 2011 and references therein). Furthermore, potential interactions between the contributing factors were not systematically taken into account.

On the other hand, recent empirical investigations treat the “exceptions” in traditional approaches as valid data and model the variability as the output of interactions between factors. Studies based on dictionary pronunciations (Zuraw, 2011), paper-based surveys (Ito, 2014 on Yanbian Korean; S. Kim, 2017), and experiments on new compound formation (Ito, 2014) have reported some consistent tendencies. The most comprehensive analysis was carried out by Zuraw (2011) on consonantal insertion in noun-noun compounds, including tensification and also /*n*/-insertion which is arguably an independent process from compound tensification (Ito, 2014).^2^ Zuraw (2011) constructed probabilistic models using more than 26,000 compounds in dictionaries and argued that a range of processing (e.g., compound frequency, word length), morphological (e.g., branching structure), and phonological (e.g., W1-final or W2-initial segment type, presence of a tense consonant) factors, and etymology (i.e., whether each constituent is a native Korean noun) had variable effects on the probability of tensification. Ito’s (2014) survey on 1,171 native compounds with 35 native speakers of Yanbian Korean, which is spoken in north-eastern China, reported similar findings to Zuraw’s (2011); high familiarity ratings and short stem length were associated with high tensification rates, while a tense consonant in a compound tended to suppress tensification. In addition, tensification was more likely to occur following an obstruent coda than a liquid or a vowel, and less likely to occur when aspirated consonants were present in the compound. Ito (2014) also carried out *wug* tests where participants identified whether the W2 onset was a lenis or tense consonant in nonce compounds in their own pronunciation. The *wug* test results replicated the survey findings related to the W1 coda type and that the presence of a tense or aspirated consonant reduced the rate of compound tensification, showing that native Korean speakers generalize the tendencies found in the existing lexicon. However, the constituent length effect (i.e., shorter compounds are more likely to undergo tensification than longer words) was not clear in the *wug* test results. Finally, S. Kim (2017) conducted a survey with 304 native Korean compounds with 21 native Seoul Korean speakers and reported the effects of word frequency, the W1 final-segment type (i.e., tensification is more likely to occur after an obstruent than a nasal or a liquid), the W2 initial onset segment type (i.e., a coronal onset is more likely to be tensified than a non-coronal onset), and the presence of a tense or aspirated consonant. Unlike in Zuraw (2011) and the survey in Ito (2014), however, S. Kim (2017) did not find a significant effect of word length.

A consensus from Zuraw (2011), Ito (2014), and S. Kim (2017) is that compounds are not dichotomously classified into those that can or cannot undergo tensification and there are between-speaker differences. To summarize their findings, the significant factors included morphological branching structure, etymology, word frequency, the compound’s familiarity to speakers, stem length, the presence of a tense or aspirated consonant in the compound, the coda type of the W1-final syllable, and the W2-initial syllable onset type (i.e., coronal vs. non-coronal). In general, compounds with a native origin, high frequency, high familiarity, a short stem, and the absence of tense or aspirated consonants led to higher tensification rates. As far as phonological context is concerned, tensification seemed most likely to occur when the W1-final syllable had an obstruent coda followed by a coronal onset in the W2-initial syllable. Zuraw (2011), Ito (2014), and S. Kim (2017) examined the effects of word frequency and compound familiarity, which were not systematically investigated in traditional approaches. In particular, the word-frequency effect suggests that language users have knowledge about the a priori distribution of tensification (cf. Ernestus, 2014; Jurafsky, 2003; Jurafsky et al., 2001; Pierrehumbert, 2001, 2002, 2006).

However, empirical approaches are still at an early stage, and previous studies have had limitations. First, because the phonological context was not tightly controlled or analyzed in detail, its effect is still not clear. The compounds in the works of Zuraw (2011), Ito (2014), and S. Kim (2017) varied in syllable structure and segmental composition. Second, these studies restricted their scope to partial variability. Zuraw’s (2011) analysis was on dictionary pronunciations which do not show between-speaker variation. Ito (2014) and S. Kim (2017) restricted their paper-based survey items to native Korean compounds. Furthermore, there has been little attempt to directly examine native Korean speakers’ speech production and we still do not have information on phonetic realizations. The previous findings concerned participants’ metalinguistic judgments not the direct output of online phonological processing. Probably part of the reason for limiting the studies’ scope lies in the challenge in dealing with the trade-off between designing a comprehensive experiment and keeping it to a manageable size. It is simply not feasible to construct an experiment to test all factors identified in previous studies. Finally, although Ito (2014) examined the generalizability of tensification with nonwords, the participants were native speakers of Yanbian Korean in China which has distinctive phonological properties and writing conventions from Korean used in South Korea. Yanbian Korean has a lexical pitch accent system unlike the standard variety, and it shows morpho-syntactic similarity to the varieties spoken in North East of the Korean peninsula (Barnes-Sadler & Yeon, 2019).^3^

One way to initiate an experimental investigation on a highly complex phenomenon is to control some factors while systematically varying others to focus on their effects. The priority of the present study was to control the phonological environment. The scope of this study was restricted to trisyllabic and tetrasyllabic compounds with a sequence of a vowel and a lenis consonant at the W1-W2 boundary (e.g., /nɑlu/ “dock” + /pɛ/ “boat”, /nɑlupɛ/ → /nɑlup*ɛ/ “ferryboat”). This is the only context where tensification can be orthographically marked. Following the standard writing conventions, *sai-sios* /sɑisiot/ (“between-ㅅ”) may be written between W1 and W2 only in native Korean compounds (e.g., 나루 *nalwu* /nɑlu/ “dock” + 배 *pay* /pɛ/ “boat” is 나룻배 *nalwuspay* /nɑlup*ɛ/ “ferryboat” not 나루배 *nalwupay*).^4^ However, the writing conventions are not strictly followed in informal writing and native Korean speakers may or may not write *sai-sios.*

To summarize, compound tensification involves a phonemic categorical change that native Korean speakers are aware of, but phonological rules alone cannot predict its occurrence. The remainder of this paper is as follows. Section 2 discusses the W2 onset duration as a dependent variable and the factors investigated in the present study, the word’s etymology, word length, W2 onset segment, speaking rate, the presence of an aspirated or tense consonant in the compound, the compound’s plausibility, and word frequency, together with the relevant hypothesis. The experiment reported in Section 3 investigated how variability in tensification probability and the consonantal constriction duration were determined by the above-stated factors. Twelve native Seoul Korean speakers were asked to memorize two compounds and produce them at two speaking rates (normal vs. fast). Section 4 discusses the experimental findings and Section 5 presents the conclusion.

## 2 The study design and factors investigated

The experimental factors were W1 Etymology (Etymology–Native vs. Loan), W2 Length (Length–Monosyllabic vs. Disyllabic), Type of W2 Onset Consonant (W2 Onset–Bilabial, Alveolar, Velar), and Speaking Rate (Normal vs. Fast). Among these, Length and W2 Onset were useful for introducing variation in the experimental materials. Repetitions of the same structure, for example, the same word length, in the experimental materials may bias participants’ production of the word boundary to be in a constant position across the compounds, which was not desirable. The priority in the experimental material design was to control the phonological context. The feasibility of experimental control was also taken into account when selecting the experimental factors. For instance, it was not feasible to cross two potential factors when designing the experimental materials, the word’s etymology and the presence of an aspirated consonant in the compound. Compared to loanwords (e.g., /kʰʌpʰi/ “coffee,” /pɛltʰɯ/ “belt”), aspirated consonants are less likely to be found in native words. Instead, the presence of an aspirated or tense consonant was incorporated as a factor in the statistical analysis. In addition, the word frequencies (W1 and W2) and native Korean speakers’ ratings of the plausibility of the target compounds were incorporated.

### 2.1 Consonantal constriction duration as a dependent variable

None of the previous studies investigated phonetic realizations of compound tensification, as it was always treated as a categorical and phonological change as judged by native speakers. The present study analyzed compound tensification as a categorical variable and also the consonantal constriction duration of the W2 onset as a gradient variable. The assumption here is that the nature of the constituent boundary is encoded in the gradient phonetic form (cf. Strycharczuk, 2019).

Cross-linguistic studies showed that phonetic properties of words with complex morphological structure are affected by word frequency, expectedness, and the constituent boundary strength (see, for instance, Bell et al., 2020; Kuperman et al., 2007; Schuppler et al., 2012 and references therein). The most relevant to the present study is Bell et al. (2020) which examined consonantal durations at the constituent boundary in English compounds (e.g., ***m*** in *stea* ***m*** *engine*, W1-final; *crea* ***m m****ini*, a geminate at the boundary; and *survey* ***m****anager*, W2-initial). Bell et al. (2020) showed that the probabilistic associations in the lexicon that speakers learned in their language acquisition process were mirrored in their phonetic outputs at the constituent boundary. To elaborate, Bell et al. (2020), in their statistical analysis, incorporated a range of frequency- and probability-related factors, such as frequency of W1 and W2, compound frequency, the probability of a given W2 for its W1, and the family size of W1 and W2 (i.e., the number of compound types with the given constituent; for instance, W1 family of *problem area* include *problem behavior* and *problem children*). The strongest effect was that the geminate consonant duration and the W1 family size were negatively correlated. In addition, across the W1-final, geminate and W2-initial conditions, the probabilistic association between the target phoneme and the W1 played a role; the more likely the target phoneme to appear with W1, the longer its duration. Bell et al. (2020) argued that these durational effects were evidence that greater contextual support for an outcome led to enhanced articulation involving lengthening. That is, when the W1 family size was large, the presence of many W2 candidates in forming a compound lowered the activation level in speakers’ lexicon, leading to short consonantal durations. On the other hand, when the probabilistic association between W1 and the W2 onset was strong, the target phoneme would be strongly activated with enhanced articulation, and therefore, produced in long durations. Finally, there was a small effect that more frequent W2s were associated with shorter duration at the boundary, indicating that more predictable, hence less informative constituents may be phonetically reduced (cf. Jurafsky et al., 2001).

For Korean, Cho (2001) showed the morphological boundary effect on articulatory timing, using electromagnetic midsagittal articulography (EMA). In the work of Cho (2001), for instance, the interval /kp/ in /pɛk.pɑl/ meaning either “(lit. white) grey hair” or “white foot” was articulated with a longer interval between two consonantal gestures (e.g., /k.p/) with higher variability when it was produced as a non-lexicalized compound (“white” + “foot”, “white foot”) compared to a lexicalized compound (“white” + “hair”, “grey hair”). This result is in line with the claim that articulatory timing relations are more stable within a lexical item than across lexical items (Browman & Goldstein, 1986). Cho (2001) also reported that the lexicalized words were more frequent and familiar to speakers, and therefore, they could be produced with greater phonetic reduction than non-lexicalized counterparts (see Conwell, 2017 and Gahl, 2008 for the frequency effect on homophones). Although Cho (2001) showed the direct effect of morphology on the phonetic form, the predictions for the present study cannot be based on his findings. All compounds in the work of Cho (2001) were disyllabic with two stops at the boundary (e.g., /kp/ in /pɛk.pɑl/) produced with two articulatory gestures (e.g., /k.p*/ following obligatory post-obstruent tensing, see Shin et al., 2013, Ch. 8). On the other hand, the present study used compounds produced with a single articulatory gesture straddling the boundary without post-obstruent tensing.

For the noun-noun compounds in the present study (see Section 3.1.2 for details), if the phonetic form is affected by probabilistic factors and/or the nature of the boundary, the factors leading to high tensification probability would also lead to an increase in the W2 onset’s constriction duration. This hypothesis is based on the assumption that Korean speakers acquire the association between the factors and the tensification which involves lengthening of consonantal duration (see Section 1.1).^5^ The language-specific learned association may be directly mirrored in the phonetic duration. The specific hypothesis for each factor is discussed below.

### 2.2 Etymology

Korean words are broadly classified into native words, Sino-Korean words and loanwords. Native Korean words and Sino-Korean words are not always subject to the same phonological processes (see Shin et al., 2013, Chs. 8–10; and see Itô & Mester, 2015 for a discussion of similar lexical strata in Japanese), while accounting for phonological adaptation of loanwords is yet another matter (e.g., Kang, 2003). It is not clear why the dual phonological systems for Sino-Korean and native words have not merged over time. One possibility is that the distinctive segmental compositions of the words in different etymological classes help speakers maintain separate systems. A role may also be played by the contextual difference that Sino-Korean words are commonly used in formal, technical, or academic contexts (see Sohn, 1999, Ch. 5), whereas native words are more frequently used in daily communication.

Native Korean words are more likely to undergo compound tensification than Sino-Korean words (Zuraw, 2011). It is probable that tensification was initially applicable to native words and spread to non-native words (see Ito, 2014 for a summary of the historical development). On the other hand, no previous study has specifically examined tensification in loanwords. Although the number of compounds formed with a loanword is increasing, even frequently used new compounds are not immediately included in dictionaries or corpora, and they have been excluded in previous studies.

In this study, W1s were either native words or loanwords. All loan W1s in the experimental materials were expected to be familiar to native Korean speakers, but the compounds with a loan W1 would be perceived as more novel and unconventional than those with a native W1. The loan W1s tended to refer to foreign but common objects (e.g., /kʰʌpʰi/ “coffee,” /pɛltʰɯ/ “belt”). Based on the findings that tensification rates were higher for native and frequent words than for non-native and infrequent words, tensification probability is hypothesized to be higher and the consonantal constriction duration at the boundary longer for native W1s than for loan W1s.

### 2.3 Word length

Tensification seems more probable in shorter than in longer compounds (Ito, 2014; Zuraw, 2011). Therefore, the same tendency is expected in the present study: higher tensification probability and longer consonantal constriction duration are predicted for monosyllabic than for disyllabic W2s. However, the shorter word length *per se* can cause lengthening of segmental duration due to polysyllabic shortening (see Fletcher, 2010 for an overview, and Jeon, 2015 for Korean). The same segment may become progressively shorter when an additional syllable is added to the word.

The length effect was not generalized to nonce words in the work of Ito (2014), and its source is not clear. Ito (2014) suggested that prosodic grouping may play a part. For instance, speakers may construct more than one APs in a long compound, and an AP boundary suppresses compound tensification in Yanbian Korean, which has lexical pitch accent. A compound carries only one pitch accent in Yanbian Korean, for example, /pɑtɑóli/ “guillemot” (/pɑtɑ́/ “sea”+ /óli/ “duck”). When a long compound is formed, there may be a conflict between the tendency to avoid having two successive syllables without pitch accent and the tendency to chunk the compound into small APs. For example, when /múl/ “water” and /otɕíŋʌ/ “cuttlefish” is combined, the output could be either /mulotɕíŋʌ/ or /múl#otɕíŋʌ/ (# indicates the AP boundary) “live cuttlefish” with tensification suppressed. The present experimental task was designed in such a way that speakers would produce one compound as one AP to avoid a confounding effect of prosodic grouping.

### 2.4 Type of W2 onset consonant

In the present study, W2 onsets were restricted to /p, k, tɕ/ for a practical reason in the target selection process (see Section 3.1.2). The effect of the W2 onset type—whether particular lenis consonants are more likely to be tensified than others—has not been thoroughly studied. S. Kim (2017) showed an interaction between the W1 coda type and the W2 onset type such that when the W1-final syllable had a liquid coda, coronal W2 onsets were tensified more frequently than non-coronal onsets in native Korean compounds. Tensification following a liquid coda is not relevant to the present experiment where all W1-final syllables were open syllables.

The segmental-type effect is intrinsically tied to the word’s frequency, as phonemes which frequently appear in frequent words have high frequencies. In light of the word-frequency effect reported in the work of Zuraw (2011), the frequency of the W2 onset type is hypothesized to affect the probability of tensification, with more frequent onset types leading to higher probabilities than less frequent types. The predicted probability of tensification by W2 onset is in the order /k/ > /tɕ/ > /p/. Out of the 18 Korean consonants, /k/ is the most frequent phoneme as a syllable onset, followed by /tɕ/ and /p/, in dictionaries and in spontaneous speech (Shin et al., 2013, Ch. 6). The same order was found in word-initial position in H. Kim’s (2005) corpus; /k/ (*n* *=* *8,558*, 13%) was more frequent than /tɕ/ (*n* *=* *7,462*, 12%) and /p/ (*n* *=* *5,513*, 9%). For consonantal constriction duration, the results cannot be directly compared across segmental types with different intrinsic duration. When the constriction duration of /tɕ/ includes the acoustic intervals for the firm closure between the articulators and the frication release of the affricate, the constriction for /tɕ/ would be longer than that for /p/ or /k/. When Yun (1998) measured the constriction duration in limited contexts (e.g., in isolated words or in a short utterance), /tɕ/ was always longer than lenis stops, and the closure durations for /p/ and /k/ overlapped. This study examines whether other factors have a constant effect across the segment types and whether there is any interaction effect, rather than the main effect of the W2 onset type on duration.

Although the frequency of tense consonants is another potential contributor, the available data were not suitable to assess this possibility. For example, in the work of Shin et al. (2013, Ch. 6) reporting the frequency of all consonants and semivowels, the tense consonants were overall much less frequent that the lenis counterparts. In their dictionary data, the frequency as a syllable onset was in the order tɕ* (1%) > k * (1%) > p* (0.4%), and in the spontaneous speech data, it was in the order k* (1.32%) > tɕ* (0.87%) > p* (0.29%). Both Shin, et al. (2013) and H. Kim (2005) analyzed the frequency of phonemes, not their phonetic realizations, and the frequency of tense realizations in speech was probably underestimated.

### 2.5 Speaking rate

As mentioned in Section 1.2, scholars have remarked that tensification marks the constituent boundary strength within a compound. However, the nature of boundary strength has never been clarified. One possibility is that the boundary strength—which can be expressed on a scale, as the term “strength” implies—is purely abstract; speakers somehow judge the strength based on their prior knowledge about the compound in their speech planning stage, and tensification is not influenced by performance factors. If this is the case, speaking-rate variation will not affect the abstract boundary strength and the categorically classified tensification, while increasing speaking rates will shorten segmental durations in general (e.g., Byrd & Tan, 1996; Kirchner, 1998). Alternatively, boundary strength may be physically adjustable; in this case, speaking rate variation will affect the categorically classified tensification. However, that fast speaking rate could join two constituents tightly leads to contrasting predictions. First, the tight joining may lead to an increase in tensification. The second possibility is that fast speaking rate causes a decrease in constriction duration, causing a decrease in tensification, because, by definition, tensification is fortition rather than lenition or reduction.

### 2.6 Presence of a laryngeally marked consonant

The presence of an aspirated or tense consonant in either W1 or W2 is known to suppress compound tensification (see Ito, 2014, on the discussion on its similarity to *rendaku* and the obligatory contour principle). Zuraw (2011) showed the suppression effect of a tense consonant, while Ito (2014) showed the effect of both an aspirated and tense consonant in both existing compounds and nonsense compounds in Yabian Korean. Ito (2014) suggested a laryngeal co-occurrence restriction with an acoustic feature [long non-modal voicing] that the co-occurrence of the two laryngeally marked consonant categories, either an aspirated or tense consonant, is blocked. S. Kim (2017) also reported a suppression effect of the laryngeally marked consonant in W2, while for W1, the effect was clear only when there were more than one marked consonants.

Therefore, a higher probability of tensification and longer consonantal constriction duration is expected in compounds without a laryngeally marked consonant compared to those with one. The laryngeal classification is straightforward for stops and affricates, but less so for fricatives. For the two denti-alveolar fricatives in Korean, /s*/ is classified as a laryngeally marked tense, but the classification of /s/ is ambiguous. The non-tense /s/ shows articulatory tension (e.g., linguopalatal contact and frication duration) similar to the stops in the lenis category but the properties related to the glottal configuration (e.g., aspiration) are similar to those of the aspirated stops (C. B. Chang, 2013). There is one glottal fricative /h/ in Korean which is associated with high F0 in the following vowel, like laryngeally marked stops (S.-A. Jun, 1996, 1998). However, the glottal fricative can be voiced intervocally like the lenis stops or deleted (e.g., Choi et al., 2006; Kang & Lee, 2019; Um, 2014). In the present study, /s/ and /h/ were classified as laryngeally unmarked consonants following Ito (2014) and S. Kim (2017).

### 2.7 Word frequency and the compound’s plausibility

The frequencies of W1 and W2 and native Korean speakers’ plausibility ratings of the target compounds were incorporated in the analysis, although they were not part of the experimental manipulation. A higher probability of tensification and longer constriction duration was expected in compounds with a frequent W1 and W2 than those with a less frequent W1 and W2 (Ito, 2014; S. Kim, 2017; Zuraw, 2011). This could suggest that in frequent words, tensification has been stabilized and structuralized, leading to reduced variability across speakers (e.g., Bybee, 2001, p. 12; Pierrehumbert, 2001, 2006). The word-frequency information in this study is from H. Kim (2005), which is the only publicly available corpus showing lemma frequency. Ratings on plausibility were chosen as a factor over familiarity, which was measured in Ito (2014). It was postulated that participants would judge familiarity based on the perceived usage frequency, while what was of interest here was whether the target compounds including a loanword were likely compounds regardless of their usage frequency. Assuming that native Korean speakers treat high-frequency or highly plausible compounds as “tighter” compounds compared to those with low frequency or plausibility, the compound’s plausibility is hypothesized to have a similar effect to word frequency.

To recapitulate the specific hypotheses, first, the probability of tensification is hypothesized to be higher when W1 is a native word (Section 2.2), and also when W2 is monosyllabic (Section 2.3). The probability of tensification is hypothesized to be affected by the W2 onset type (Section 2.4). If boundary strength is abstract and not influenced by speaking-rate variation, then an increase in speaking rate will not affect the tensification probability. Alternatively, if categorical tensification is affected by physical adjustment, then there are two possibilities for the outcomes. Fast speaking rate could lead listeners to join W1 and W2 tightly, leading to an increase in tensification, or cause durational reduction, causing a decrease in tensification (Section 2.5). The presence of a laryngeally marked consonant in a target compound is hypothesized to reduce tensification probability (Section 2.6). Finally, an increase in word frequency and plausibility ratings is hypothesized to increase the probability of tensification (Section 2.7).

For the W2 onset constriction duration, the factors associated with high tensification probability, the native word, the absence of a laryngeally marked consonant, high word frequency, and high plausibility ratings are hypothesized to have a lengthening effect. The word length, the W2 onset type, and speaking rate are expected to affect duration, but their effects would be irrelevant to tensification.

## 3 Experiment

### 3.1 Methods

#### 3.1.1 Participants

The participants were 12 native Korean speakers who were 18–30 years old without a history of speaking or hearing impairments (six female, six male, age *M* *=* *24, SD* *=* *1.7*). Only those who had lived outside Korea for less than 2 years were recruited. Recruitment was done via online advertisement. One participant had lived in the United States for 1 year, but the rest had not lived outside Korea for more than 2 months. All participants had been brought up and educated in Seoul and the surrounding areas, speaking the standard Seoul variety of Korean. The study was approved by the local ethics committee of the University of Central Lancashire (BAHSS 447).

#### 3.1.2 Selection of experimental targets

The experimental targets were 36 pairs of noun-noun compounds which consisted of two words (W1 and W2). The members of each compound pair differed in W1 etymology (native vs. loan) but shared a native W2, for example, /tɑmpɛ-kɑp/ “cigarette price” (/tɑmpɛ/, CVC.CV native W1 “cigarette” + /kɑp/ “price”) versus /tʰɛksi-kɑp/ “taxi fare” (/tʰɛksi/, CVC.CV loan W1 “taxi”+ /kɑp/ “price”). The design of the target compounds was: 2W1 Etymology (Native vs. Loan) × 2W2 Length (Monosyllabic vs. Disyllabic) × 3W2 Onset Type (Bilabial, Alveolar, and Velar) × 6 items for each W2 Onset Type (see Appendix for the list of all target compounds). All W1s were disyllabic and they were combined with either a monosyllabic or disyllabic W2 to create a trisyllabic or tetrasyllabic compound. The syllable structure of W1 in each pair was controlled as much as possible, but control was not always feasible while avoiding creating a semantically awkward compound. There were five pairs which did not have matching syllable structures. In four out of the five pairs, one of the compounds had a semivowel (e.g., /tɕʰimɑ-tɕulɯm/ “skirt gathering” vs. /sjʌtɕʰɯ-tɕulɯm/ “shirt wrinkles”). In one pair, the W1-initial syllable structure differed between /kuksu-tɕukʌk/ “noodle spatula” (CVC) and /iŋkʰɯ-tɕukʌk/ “ink spatula” (VC).

In selecting the target compounds, the priority was to control constituent length, syllable structure and segmental composition while ensuring that none of the target compounds was semantically awkward. The target selection procedure was as follows: first, the author identified trisyllabic or tetrasyllabic noun-noun compounds with a W2-initial syllable onset in the corpus of frequently used nouns in written Korean (63,836 nouns in H. Kim, 2005). Proper nouns and compounds with a W1 that cause aspiration in the W2 onset consonant (and therefore cannot undergo tensification) were excluded (e.g., /ɑm-twɛtɕi/ “female pig” realized as /ɑmtʰwɛtɕi/). In the corpus, only 19 compounds (18 native and 1 loan compounds) were identified as appropriate for achieving the desired structural controls. W1 and W2 candidates which could form a plausible compound were selected separately from the corpus to add more target compounds. When selecting W2s with different W2 onset types (bilabial, alveolar, and velar), the bilabial and velar W2s were stop-initial, whereas the alveolar W2s were affricate-initial because the majority of the W2 candidates with an alveolar stop resulted in semantically awkward compounds. An additional 24 compounds (12 trisyllabic and 12 tetrasyllabic) were selected as practice materials. A summary of the frequency statistics of W1s and W2s is provided in Table 2. Overall, native W1s were higher in frequency compared to loan W1s, while the frequency trends of other experimental factors were not clear. The frequencies of the target compounds were not incorporated in the analysis, since 53 target compounds out of 72 were not found in H. Kim’s (2005) corpus.

**Table 2.** Frequency Statistics and Plausibility Ratings (Log-transformed) for Compounds by Etymology, W2 Length, and W2 Onset Groups.

Length	W2 onset	W1 frequency		W2 frequency		Plausibility	
		*M*	*SD*	*M*	*SD*	*M*	*SD*
Native							
Mono	Bilabial	3.15	1.49	6.1	0.53	1.44	0.11
	Alveolar	3.8	2.41	4.68	3.11	1.42	0.17
	Velar	6	1.01	4.72	2.7	1.42	0.13
Di	Bilabial	3.34	1.45	3.97	1.14	1.3	0.34
	Alveolar	4.34	0.56	4.19	2.12	1.24	0.31
	Velar	4.33	1.76	3.45	1.79	1.26	0.34
Loan							
Mono	Bilabial	1.92	1.67	6.1	0.53	1.09	0.4
	Alveolar	1.92	1.03	4.68	3.11	0.78	0.47
	Velar	3.18	2.23	4.72	2.7	1	0.38
Di	Bilabial	3.27	2.12	3.97	1.14	1.14	0.11
	Alveolar	3.36	0.71	4.19	2.12	1.05	0.23
	Velar	3.6	2.55	3.45	1.79	1.19	0.32

Note that the W2 was shared in a native and loan compound pair and, therefore, W2 frequency is the same between the native and loan conditions.

The majority of the target compounds were semantically transparent. That is, the compound’s meaning was consistent with the constituents’ meaning (e.g., *carwash*, see Pollatsek & Hyönä, 2005, for transparency and opacity). However, it was not feasible to control the degree of transparency across the target compounds while not overly complicating the experimental design. Some target compounds were lexicalized and frequently used but not semantically transparent (e.g., /mʌli-kɯl/ “introduction,” lit. “head-text”). There were also some semantically ambiguous cases. For instance, the target compound pair /nolu-tɕɑm/ “roe-deer sleep” and /pipʌ-tɕɑm/ “beaver sleep” can be interpreted as transparent compounds, but in Korean /nolu-tɕɑm/ could be opaque (the compound’s meaning not being consistent with the constituents’ meaning, e.g., *pineapple*), referring to a “light sleep” whereas /pipʌ-tɕɑm/ does not have the opaque counterpart.

The target compounds were classified into two categories depending on whether either W1 or W2 contained a laryngeally marked aspirated or tense phoneme. In this process, the form after an application of the obligatory post-obstruent tensing rule (see Shin et al., 2013, Chs. 8) was used for two compounds which had a sequence of two lenis consonants, /pɑksɯ-tɕokɑk/→/pɑks*ɯ-tɕokɑk/ “box piece (loan W1)” and /nɑksi-pɛ/→/nɑks*i-pɛ/ “fishing boat (native W1).” Eleven target compounds with native W1 and 27 with loan W1 had a laryngeally marked consonant. Only six of them had a tense consonant. Therefore, the 39 compounds were classified to have a laryngeally marked consonant for analysis, instead of being categorized into “aspirated” and “tense” subsets.

An online survey was conducted to collect the plausibility ratings of the target compounds (5: *highly plausible*, 1: *not plausible at all, rating M* *=* *3.47, SD* *=* *1*). Participants were 12 native Korean speakers (age *M* *=* *37.12* *years, SD* *=* *2.23*) who were recruited by advertisement on various social media networks. The plausibility ratings were higher for W1 Etymology–native than for loan, but no other clear trends were associated with other experimental factors (Table 2, see text in Supplementary Materials for procedure).

To validate the reliability of the word-frequency statistics and the relationship between word frequency and plausibility, correlations between the numbers of search results per second for the exact match for W1s, W2s, and the target compounds on the Google search engine (searched on December 16, 2019) and the W1 and W2 frequencies, and plausibility rating were obtained. The Google search engine collects information on the word’s usage on the internet regardless of context, and the number of search results can be an effective frequency measure. The count for the whole compounds was calculated as the sum of the counts for the compounds with and without *sai-sios* in the written form, since native Korean speakers may or may not use *sai-sios* in writing, as explained in Section 1.

All frequency measures and plausibility ratings showed a skewed distribution and were therefore log-transformed. The correlations are summarized in Table S1 (Supplementary Materials); W1 frequency and plausibility ratings showed a positive correlation with the Google search results. The results show that the statistics from H. Kim’s (2005) corpus were reasonably reliable indicators of the word’s usage frequency. W2 frequency did not correlate with the compound counts or plausibility rating. The reason for this is probably that there were fewer target W2s than W1s, and the W2s were carefully selected to systematically differ in their W2 onset consonant type. The results from Google search were not incorporated into the statistical analysis, since they were correlated with the frequency statistics and plausibility rating.

#### 3.1.3 Experimental materials and procedure

The experimental materials were 36 lists of two compounds each: one trisyllabic compound and one tetrasyllabic compound, for example, /nɑksipɛ/, /pɑkʰwitɕɑkuk/, “fishing boat”, “wheel trace.” Half of the lists had a trisyllabic compound first and the other half had a tetrasyllabic compound first. The target compounds were presented in lists rather than individually to lead participants to produce each compound as an AP. All compounds were written in the Korean script without *sai-sios*, which participants could interpret as indicating compound tensification.^6^ In the Korean script, letters are combined into a syllabic block; trisyllabic compounds were always written in three blocks, and tetrasyllabic compounds in four blocks.

The experiment took place in a sound-attenuated room at the Hanyang Phonetics and Psycholinguistics Laboratory, Hanyang University, Seoul. All instructions to participants were given in Korean by the experimenter, who is a native Seoul Korean speaker. The recording was carried out with a TASCAM HD-P2 digital recorder at 24 bit with a 44.1 kHz sampling rate and a Shure KSN44A microphone placed on a desk-stand. The recordings were saved in wav format. Prior to the experiment, all participants filled in a consent form. One participant was recorded at a time, sitting in front of a laptop and a microphone. At each trial, the participants were instructed to memorize the two-compound list appearing on the screen. After 5 seconds the written list was automatically replaced by written instructions prompting the participants to start speaking. In each trial, the participants were asked to repeat each list twice into the microphone. When the recording was completed, the participants pressed the spacebar on the keyboard to proceed to the next trial. They were also asked to proceed by pressing the spacebar if they could not remember the target words. This memory task was used to keep the participants from simply reading the text written without *sai-sios*. It was expected that the memory task would lead them to internalize the phonological forms and produce their own phonetic coding.

There were two speaking-rate blocks. The participants were asked to speak at a comfortable rate in the first block, but as fast as they could in the second block. In each block, a practice session preceded the main experiment, and the presentation order of the lists was randomized for each participant. Each list appeared twice in each block. In the main experiment, each participant produced 576 tokens (2 target compounds × 36 lists × 2 readings per trial × 2 repetitions of each list × 2 speaking-rate blocks). Therefore, the total number of data points to be collected was 6,921 (2 W1 Etymology × 2 W2 Length × 3 W2 Onset × 6 W2 items × 2 Speaking Rate × 2 within-trial repeats × 2 within-block repeats × 12 participants). Psychopy 2 ver. 1.85.1 (Peirce et al., 2019) was used for the stimulus presentation.

### 3.2 Acoustic and auditory analysis

**Figure 1.**
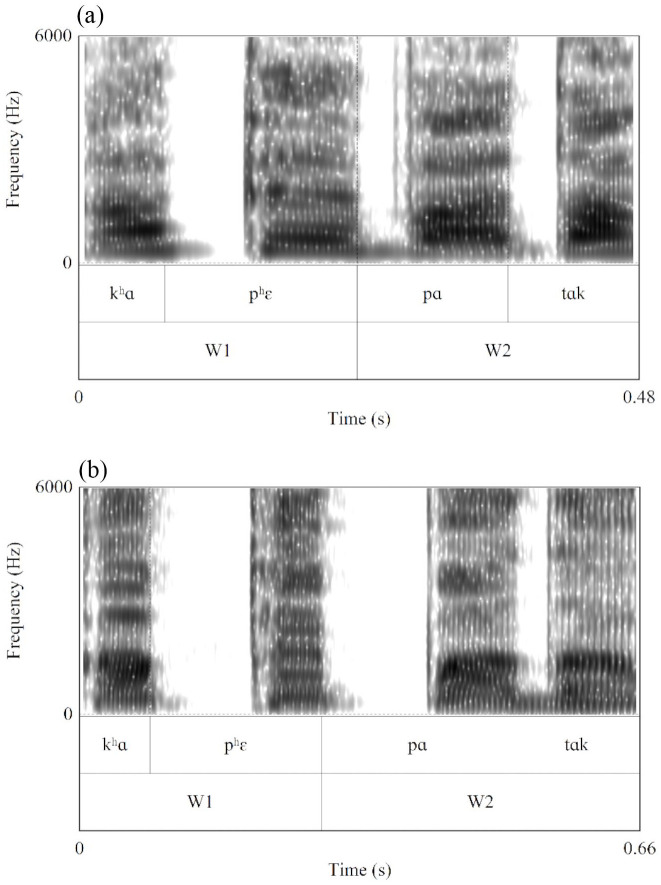
Spectrograms with the syllable and constituent word boundaries annotated in /kʰɑpʰ́ɛpɑtɑk/ “café floor.” In (a), the W2 onset consonant is realized as a lenis stop /p/ with voicing throughout the closure. In (b), it is realized as a tense stop /p*/ with a long closure without voicing.

The recorded compounds were acoustically and auditorily examined using Praat ver. 6.0.37 (Boersma & Weenink, 2018). The author, a trained phonetician and native speaker of Seoul Korean, classified the W2 onset consonant in each compound based on simultaneous auditory judgment and visual inspection of spectrograms. The W2 onset was classified as either a lenis (e.g., /nɑksi-pɛ/ “fishing boat” realized as [nɑks*ipɛ] or [nɑks*ibɛ]) or tense (e.g., [nɑks*ip*ɛ]). The perceived category was clear in general with accompanying acoustic cues. For instance, a tense stop was identified by a long consonantal closure duration and irregular pulses at the onset of the following vowel, and a lenis stop by short closure and breathiness in the following vowel (see Table 1 and Figure 1). Another native Korean speaker, a postgraduate student in phonetics, carried out the classification by ear with 1,340 randomly selected samples (20%) out of 6,633 recorded compounds. The agreement between the two raters was high at 98%. The author re-examined the tokens where there was disagreement. These tokens had conflicting acoustic cues, such as partial voicing, which often appears in an intervocalic lenis consonant, in relatively long W2 consonantal closure, which is typical for a tense consonant. Following examination, a decision was made that the categorization of tokens where not all the relevant cues were present would be based on the acoustic properties of the following vowel; that is, whether there were irregular pulses near the vowel onset and pressed voice, which are typical cues for a tense consonant. Then the author re-examined the whole dataset to ensure that consistent criteria were applied.

The duration of various acoustic intervals was measured from spectrograms. The boundaries of consonantal closure, aspiration, frication noise for affricates, and the compound were annotated in the Praat textgrids (see Turk et al., 2006 for segmentation criteria). The first and last consonantal intervals of each compound, which were closure in a stop or affricate, the frication portion in a fricative, a nasal and a liquid, were excluded in annotation because their onset or offset could not be reliably identified. Therefore, the compound duration was measured from the release of the onset consonant in the first syllable to the compound-final vowel offset. In velar stops with multiple bursts, the time of consonantal release was marked at the first burst. The vowel onset and offset were, respectively, marked at the beginning and the end of a voicing bar and F2. When a vowel was completely devoiced without clear formant structure and a voicing bar following a voiceless fricative, the devoiced portion was included in the preceding fricative.

### 3.3 Statistical analysis and results

#### 3.3.1 Tensification rates across speakers and compounds

**Figure 2.**
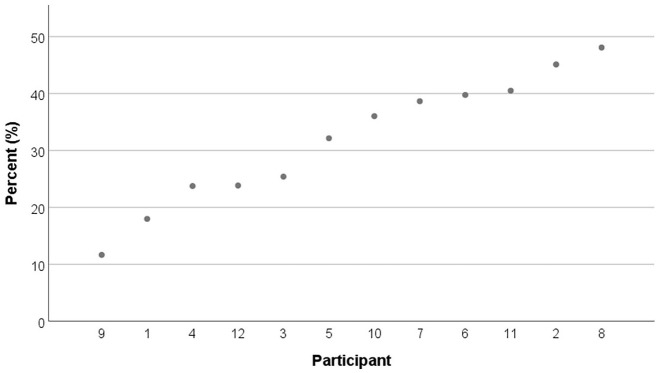
Averaged compound tensification rate (%) for each participant (participants 1–12) in ascending order.

**Figure 3.**
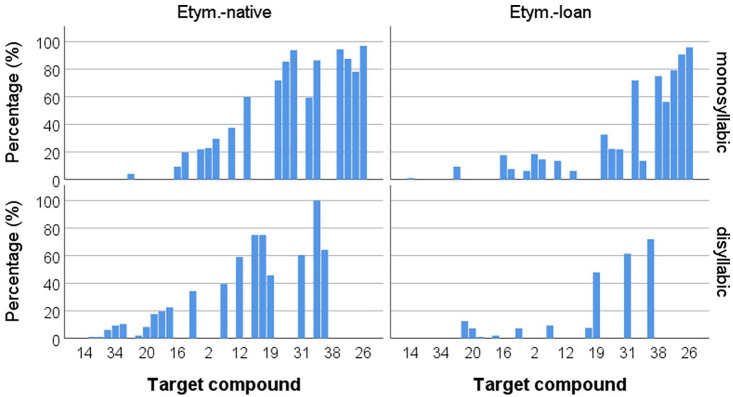
Average compound tensification rate (%) for each target compound (*x*-axis) in ascending order by W2 Length (Monosyllabic vs. Disyllabic) and W1 etymology (Native vs. Loan).

The compounds realized with a categorically ambiguous W2 onset consonant with long closure (e.g., /nɑksi-pɛ/ as [nɑks*itpɛ] “fishing boat,” *n* *=* *14*, 0.2% of the total number of data points) were excluded and 6,619 data points were analyzed. In total, 32% (*n* *=* *2,108*) of the compounds underwent tensification. Figures 2 and 3 show the tensification rates across the participants and the target compounds. Specifically, Figure 2 shows that the compound tensification rates varied between 10% and 50% across the participants; the rates presented in the ascending order show a continuous distribution. Figure 3 also shows a continuous distribution of the rates across the target compounds in general. In Figure 3, the compounds for Etymology–Loan and W2 Length–Disyllabic (i.e., those with a loan W1 followed by a disyllabic W2) showed relatively low tensification rates when presented by Etymology and W2 Length. The rate distributions in Figure 3 showed that, overall, more compounds underwent tensification for Etymology–Native than for Loan and also for W2 Length–Monosyllabic than for Disyllabic.

#### 3.3.2 Statistical analysis: tensification as a categorical variable

Mixed-effect logistic models were fitted to the data using the *glmer* function in the lme4 package (Bates et al., 2015) with R ver. 4. 0. 3 (R Core Team, 2019). The models estimated the maximum likelihood of the positively coded compound tensification (0: lenis realization, 1: tense realization; *n* *=* *6,619*, ambiguous tokens excluded). Initially, a full model was fitted with fixed factors, W1 Etymology (Etymology–Native, Loan), W2 Length (Length–Monosyllabic, Disyllabic), Type of W2 Onset Consonant (W2 Onset–Bilabial, Alveolar, and Velar), Speaking Rate (Normal, Fast), Presence of Laryngeally Marked Consonant, W1 Frequency, W2 Frequency, Plausibility Rating, and all interaction terms including the eight-way interaction and lower-order interactions with random intercepts for participants and items. The full model did not converge. Then a model was constructed with all fixed factors, (1) the two-way interaction between Etymology and Laryngeally Marked which could be confounded (Section 2.2) and (2) the seven-way interaction between other fixed factors and their lower-order interactions with random intercepts for participants and items, but the convergence error persisted. From this model, a backwards stepwise procedure was used to eliminate interaction terms leading to the convergence errors one by one. In this process, the interaction terms involving W1 Frequency, W2 Frequency, and Plausibility Rating were discarded.

**Figure 4.**
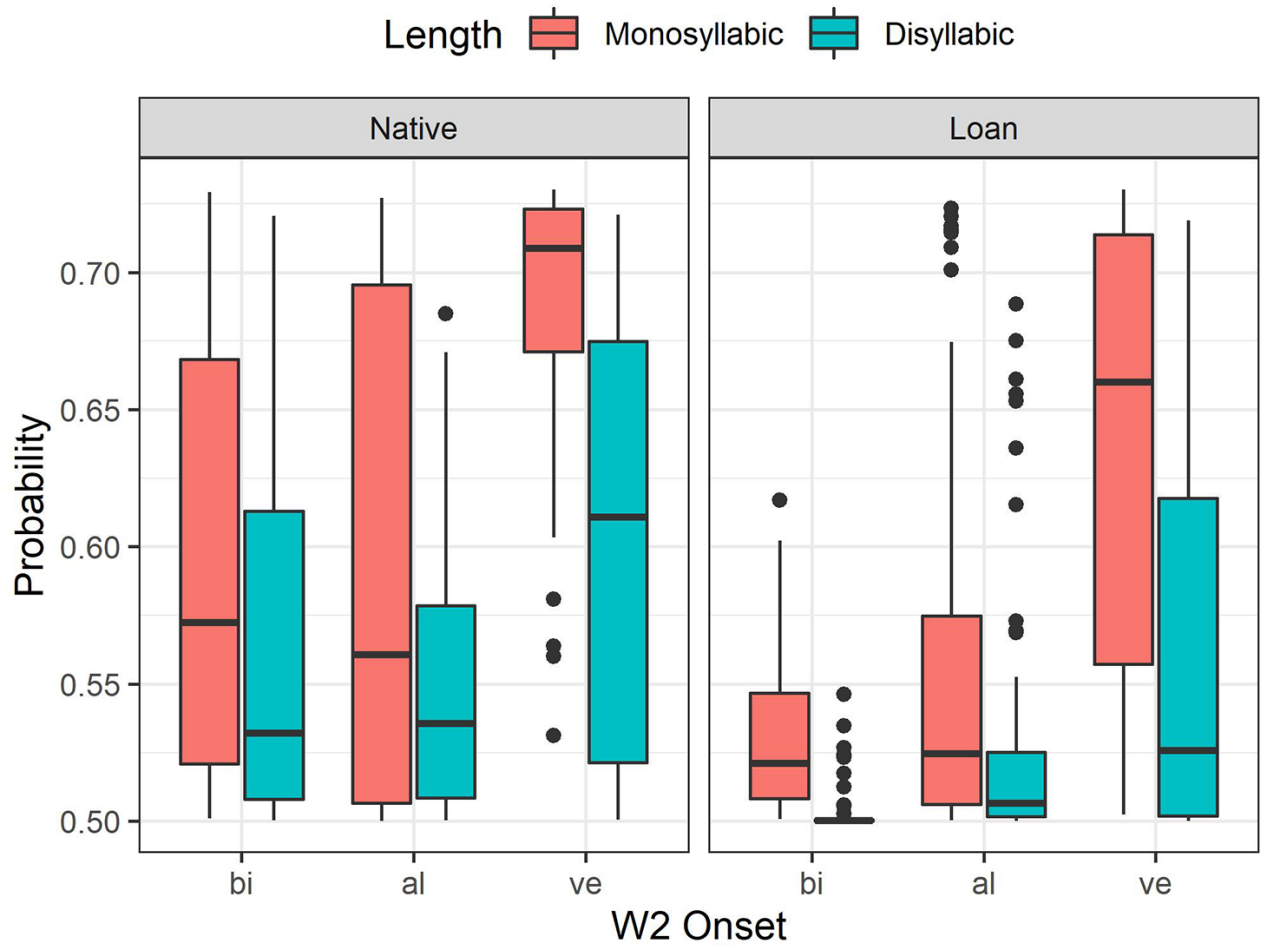
Boxplots showing the distribution of fitted tensification probability by Etymology, Length and W2 Onset (*M* = 0.5, *SD* = 0.08, *n* = 6,619). The thick line in the box indicates the median, the bottom and the top of the box indicates the 25th and the 75th percentiles, respectively. The whiskers extend to 1.5 times the height of the box, but if there are no data in this range, the whiskers show the maximum or minimum values.

The initial convergent model incorporated the fixed factors, Etymology, Length, W2 Onset, Speaking Rate, Laryngeally Marked, the four-way Etymology × Length × W2 Onset × Speaking Rate interaction term and all lower-order interactions, the two-way Etymology × Laryngeally Marked interaction term, and also W1 Frequency, W2 Frequency, and Plausibility Rating with random intercepts for participants and items. When Speaking Rate was coded as a scale variable (phonemes per second) in alternative models, they did not converge and, therefore, Speaking Rate was coded in two levels representing the two blocks in the experiment (Normal and Fast). At the fast speaking rate, speakers reduced the syllable duration to 86% of that at the normal rate for syllables with a lenis onset and to 78% for syllables with a tense onset (lenis, normal: *M* *=* *161* ms, fast: *M* *=* *139* ms; tense, normal: *M* *=* *261* ms, fast: *M* *=* *204* ms). A backwards stepwise procedure was used to prune the initial convergent model, so that, interaction terms or fixed factors remained only if they improved the model fit. For instance, comparisons were made between a model containing a particular interaction term and a model excluding this interaction term while keeping all other terms identical. A factor or an interaction term was removed when a log-likelihood test (using the *anova* function) indicated that a lower-order model had a significantly better fit (α *=* *0.05*) with a lower Akaike information criteria (AIC) value. When a convergence warning occurred, the *allFit* function was used to check whether all optimizers produced equivalent values, which verifies that the warning was a false-positive (Bates et al., 2019). The results of the log-likelihood tests are summarized in Table 3 and they are used in interpreting the effect of fixed factors together with the fitted tensification probabilities from the final model (Figure 4).

**Table 3.** Results of the Log-Likelihood Tests with Fixed Factors, W1 Etymology, W2 Onset Type, and W2 Length, Speaking Rate, Presence of Laryngeally Marked Consonant, Plausibility Rating, W1 Frequency and W2 Frequency, with Tensification as a Categorical Dependent Variable.

	χ^2^	*df*	*p*
Etym × W2 Onset × Length × Speaking Rate	1.94	3	.59
Etym × W2 Onset × Length	10.27	3	.02*
Etym × W2 Onset × Speaking Rate	7.38	3	.06
W2 Onset × Length × Speaking Rate	4.79	3	.19
Etym × Laryngeal	0.29	1	.59
Etym × Speaking Rate	1.29	1	.26
W2 Onset × Speaking Rate	0.94	1	.62
Length × Speaking Rate	2.06	1	.15
Speaking Rate	1.14	1	.29
Laryngeal	4	1	.045*
Plausibility	7.01	1	.01**
W1 Frequency	6.53	1	.01*
W2 Frequency	10.51	1	.001**

*p* < .05*, *p* < .01**, *p* < .001***.

In the final model, Etymology, W2 Onset, Length, and their two- and three-way interaction terms were retained (Table S2, Supplementary Materials), because there was a significant Etymology × W2 Onset × Length interaction effect (Table 3). Laryngeally marked, W1 Frequency, W2 Frequency, and Plausibility Rating, which had significant effects (Table 3), were also retained, while Speaking Rate, which did not have any significant effect, was not incorporated.

Only significant effects from the log-likelihood tests (Table 3) are discussed here. First, there was a significant Etymology × W2 Onset × Length interaction effect (χ^2^ *=* *10.27, p* *<* *.05*). In Figure 4, the probability distributions and medians show that the effects of Etymology and Length were consistent; the tensification probability was lower for Etymology–Loan than for Native, and also lower for Length—Disyllabic than for Monosyllabic in all W2 Onset conditions. The main source of the significant Etymology ×  W2 Onset × Length interaction effect was from W2 Onset–Bilabial and Alveolar, while Velar was consistently associated with high tensification probabilities for both Etymology–Native and Loan. For W2 Onset–Bilabial and Alveolar, the probabilities show more pronounced variation for Etymology–Native than for Loan (Figure 4). For Etymology–Native, W2 Onset–Velar led to a high tensification probability compared to Bilabial and Alveolar, and Length–Monosyllabic also increased the probability from Disyllabic. For Etymology–Native, the differences between Bilabial and Alveolar were not considerable; for W2 Length–Monosyllabic, the median for Bilabial was marginally higher than that for Alveolar, whereas for W2 Length–Disyllabic, the medians were similar between Bilabial and Alveolar, with the probability distribution showing a more negative skew for Bilabial than for Alveolar. On the other hand, for Etymology–Loan, the probabilities were in the order Velar > Alveolar > Bilabial in both Length conditions. In particular, the compounds for W2 Onset–Bilabial, Length–Disyllabic had notably low probabilities.

To recapitulate, for the Etymology–Native compounds, the variability in the tensification probability associated with W2 Onset was greater than for the Loan compounds, except for the compounds in the W2 Onset–Velar and Length–Monosyllabic condition, which led to high tensification probability in a congruous manner. On the other hand, while the hypothesized trend associated with W2 Onset was observed for Etymology–Loan, the variability was particularly low for the compounds in the W2 Onset–Bilabial and Length–Disyllabic condition. That is, the variability in the probabilities was relatively reduced in the two extreme experimental conditions when the conditions leading to high probability (i.e., Etymology–Native, W2 Onset–Velar, Length–Monosyllabic) were combined, and also when the conditions leading to low probability were combined (i.e., Etymology–Loan, W2 Onset–Bilabial, Length–Disyllabic).

The presence of laryngeally marked consonant significantly decreased tensification probability (χ^2^ *=* *10.27, p* *<* *.05*, Table 3; *est.* *=* *–1.07, SE=* *0.53*, Table S2) while it did not have a significant interaction with Etymology. Furthermore, W1 Frequency, W2 Frequency, and Plausibility Rating significantly affected tensification probability (Table 3). Their effects were consistent in that an increase in frequency and plausibility rating led to an increase in tensification probability (W1 Frequency, *est.* *=* *0.36, SE* *=* *0.14*; W2 Frequency, *est.* *=* *0.36, SE* *=* *0.11*; Plausibility, *est.* *=* *2.26, SE* *=* *0.83*, Table S2, Supplementary Materials).

#### 3.3.3 Statistical analysis: consonantal constriction duration

General linear mixed models were fitted to the data using the *lmer* function in the lme4 package (Bates et al., 2015) with R ver. 4. 0. 3 (R Core Team, 2019). First, a backward model fitting procedure as in Section 3.3.2 was applied with a perceived category of W2 onset consonant (lenis vs. tense realization) as an additional factor. Then, the perceived category turned out to be the main predictor for constriction duration (*est.* *=* *71.86, SE* *=* *1.64*), and it was also involved in a significant three-way W1 Etymology × Perceived Category × Laryngeally Marked interaction (*df* *=* *1*, χ^2^ *=* *6.61, p* *<* *.05*).

Therefore, the data were split into two sets for further modeling to examine the effects of factors on the phonetic form in each phonemic category (lenis realization, *n* = 4,511; tense realization, *n* = 2,108). Following the results reported in Section 3.3.2, the initial full model was constructed with all fixed factors, W1 Etymology (Etymology–Native, Loan), W2 Length (Length–Monosyllabic, Disyllabic), Type of W2 Onset Consonant (W2 Onset–Bilabial, Alveolar, Velar), Speaking Rate (Normal, Fast), Presence of Laryngeally Marked Consonant (Marked, Unmarked), W1 Frequency, W2 Frequency, Plausibility Rating, and the three-way interaction term W1 Etymology × W2 Onset Type × Length, their lower-order interactions, and the two-way W1 Etymology × Laryngeally Marked interaction, with random intercepts for participants and items. For model comparisons, all models were fitted by maximum likelihood.

The full results of the log-likelihood tests are provided in Table S3 (Supplementary Materials). For the “lenis” data (Speaking Rate–Normal, *M* *=* *57.42* ms, *SD* *=* *34.33*; fast, *M* *=* *47.39, SD* *=* *27.35*), the Etymology × Laryngeally marked interaction (*df* = 1, χ^2^ = 4.28, *p* < .05), W2 Onset (*df* = 2, χ^2^ = 82.21, *p* < .001), Length (*df* = 1, χ^2^ = 25.58, *p* < .001), and Speaking Rate (*df* = 1, χ^2^ = 185.25, *p* < .001) effects were significant. The final model was constructed with only the fixed factors and their interaction which showed a significant effect (*p* *<* *.05*). The just noticeable difference (JND) for segmental duration is estimated in the range of 10–40 ms (Klatt, 1976; Lehiste, 1970, p. 13). Therefore, the estimated parameter should be larger than 10 ms for a factor to have a perceivable effect. The final models (Table 4) showed that for the “lenis” data, compared to the reference level (Etymology–Native, W2 Onset–Bilabial, Length–Monosyllabic, Speaking Rate–Normal, Laryngeal–Unmarked), Etymology–Loan, W2 Onset–Velar, Length–Disyllabic, Speaking Rate–Fast, and Laryngeal–Marked decreased the constriction duration, as indicated by the negative parameter. The interaction between Etymology–Loan × Laryngeal–Marked with a positive parameter suggests that the trend could be reversed, but this effect is not likely to be perceivable under the JND. For instance, although Etymology–Loan and Laryngeal–Marked, respectively, decreased constriction duration from the reference level (intercept, est. = 58.90 ms, *SE* = 3.62), the constriction duration of a W2 bilabial onset in a compound with a monosyllabic W1 for the Etymology–Loan and Laryngeal–Marked condition was estimated to be 59.63 ms (58.90–3.91–1.94 + 6.58). Constriction duration increased from the reference level for W2 Onset–Alveolar, but none of W1 Frequency, W2 Frequency, and Plausibility had a significant effect. The parameters in Table 4 show that only W2 Onset–Alveolar, Length–Disyllabic, and Speaking Rate–Fast led to a perceivable difference.

**Table 4.** Final Models for Constriction Duration (ms) as a Dependent Variable.

	Lenis			Tense		
	Estimate	*SE*	*t*	Estimate	*SE*	*t*
Intercept	58.90	3.62	16.26	125.41	10.81	11.60
Etym–Loan	–3.91	3.35	–1.17			
W2 Onset–Alveolar	22.66	2.41	9.40	25.79	4.73	5.45
W2 Onset–Velar	–3.63	2.53	–1.43	–2.47	4.43	–0.56
Length–Di	–11.42	2.06	–5.53	–32.92	3.66	–9.00
Speaking Rate–Fast	–10.77	0.78	–13.74	–32.66	1.38	–23.76
Laryngeal–Marked	–1.94	3.13	–0.62			
Plausibility				13.88	5.72	2.43
Etym–Loan × Laryngeal–Marked	6.58	4.49	1.47			

The model was constructed separately for lenis realizations and tense realizations (tensified) of W2 consonant. *SE*: standard error.

For the “tense” data (Speaking Rate–Normal, *M* *=* *142.62* ms, *SD* *=* *43.24*; fast, *M* *=* *111.53, SD* *=* *49.07*), the effects of W2 Onset (*df* = 2, χ^2^ = 37.44, *p* < .001), Length (*df* = 1, χ^2^ = 50.54, *p* < .001), Speaking Rate (*df*= 1, χ^2^ = 496.12, *p* < .001), and Plausibility (*df* = 1, χ^2^ = 5.76, *p* < .05) were significant (Table S3, Supplementary Materials). The final model showed that (Table 4), compared to the reference level, W2 Onset–Velar, Length–Disyllabic, and Speaking Rate–Fast reduced constriction duration with a perceivable effect. On the other hand, W2 Onset–Alveolar increased constriction duration. Although W1 Frequency and W2 Frequency did not have a significant effect, an increase in plausibility was associated with an increase in constriction duration (*est.* *=* *13.88* ms, *SE* *=* *5.72*).

### 3.4 Summary

The compound tensification rates showed continuous distributions across speakers and compounds. For tensification as a categorical output, the hypotheses regarding etymology (Section 2.2) and constituent length (Section 2.3) were supported; tensification probability was relatively higher for native than for loan W1s, and also for short than for long compounds. The hypothesis regarding the type of W2 onset consonant based on phoneme frequency (Section 2.4) was partially supported in that the most frequent velar onset /k/ was associated with high tensification probability. However, there was a three-way interaction involving etymology, length and the W2 onset type in modeling the probability, while variability in tensification probability was relatively reduced in two extreme experimental conditions, which had either the highest probability (W1 Etymology–Native, W2 Onset Type–Velar, W2 Length–Monosyllabic) or the lowest probability (W1 Etymology–Loan, W2 Onset Type–Bilabial, W2 Length–Disyllabic). The speaking rate did not have a significant effect on tensification probability. The results support the hypothesis that word boundary strength linked to compound tensification is abstract and is not influenced by speaking rate variation (Section 2.5). As hypothesized, the presence of a laryngeally marked consonant reduced tensification probability (Section 2.6), while frequent W1s and W2s and high compound plausibility led to an increase in tensification probability compared to infrequent constituents or less plausible compounds (Section 2.7).

The evidence supporting the hypothesis that the factors associated with high tensification probability would also increase the W2 onset constriction duration was not strong, once the categorical output was considered. The perceived category of the W2 onset (lenis vs. tense) accounted for a large part of the durational variation in the data. For the lenis realizations, there were a significant effect of Etymology,Laryngeally marked, and an interaction effect between W1 Etymology and Laryngeally Marked, but their perceptual effects would be negligible. On the other hand, for the tense realizations, an increase in the plausibility was associated with a longer duration as hypothesized.

For both lenis and tense realizations, W1 frequency and W2 frequency did not have a significant effect. W2 Onset Type (Bilabial, Alveolar, and Velar) had a significant effect demonstrating the difference in intrinsic segmental durations. Monosyllabic W2s were associated with longer constriction duration compared to disyllabic W2s. Fast speaking rates reduced the constriction duration. Because W2 Onset Type, Length, and Speaking Rate effects were expected regardless of tensification, they are not discussed further.

## 4 Discussion

The present study investigated the realization of compound tensification, systematically manipulating the W1 etymology (native vs. loan), the place of articulation for W2 onset consonant (referred to as the “onset type,” bilabial, alveolar, velar), the W2 length (monosyllabic vs. disyllabic) of the target compounds, and speaking rates, while controlling the phonological context. Scale variables, such as word frequency and the compound’s plausibility, and also the presence of a laryngeally marked aspirated or tense consonant were incorporated in the analysis. To my knowledge, this is the first experimental investigation of compound tensification in Korean using both existing compounds and compounds formed with a loanword varying in perceived plausibility. In the experiment, native Korean speakers were asked to memorize the target compounds before recording them. There were two “speaking rate” conditions (normal vs. fast). The recorded speech data were analyzed to judge whether the W2 lenis onset was categorically tensified and to measure the constriction duration of the W2 onset. It should be noted that the nature of the experimental task could have resulted in higher variability in the phonetic outputs compared to simple tasks, such as text reading.

### 4.1 Factors affecting compound tensification

As hypothesized (see Section 2), the constituent’s etymological class, length, the onset type of the W2-initial syllable, the presence of an aspirated or tense consonant, the constituent word frequency, and the plausibility of the compound affected compound tensification. The variation in speaking rate did not have a significant effect on the categorical production of tensification (Section 3.3.2) while it was a significant predictor for constriction duration, fast rates reducing the duration (Section 3.3.3). That is, there was no evidence that speaking fast has an effect of physically tightening the boundary and consequently increasing tensification rates, or the reduction in duration under fast rates leads speakers to decreases tensification rates. In a nutshell, the “tightly joined” compounds as judged by native Korean speakers were more likely to be tensified, and the judgment was at the cognitive level, not influenced by speaking-rate variation. The findings related to each predictor are further discussed below.

First, tensification probability was consistently higher for native W1s than for loan W1s, that is, native W1s were more likely to trigger tensification of the same W2 onset than loan W1s. However, the etymology effect was not dichotomous, as shown in the continuous distribution of tensification rates (Figure 3). The etymology effect may suggest that compound tensification is spreading from native words to Sino-Korean words and loanwords. In the spreading process of tensification, variability across compounds is expected. Non-native compounds would be associated with zero frequency when introduced into Korean, and they will differ in the extent to which they are perceived as plausible or familiar by language users at a certain point in time (the effect of plausibility or familiarity is discussed below).

Second, tensification probability was higher for monosyllabic W2s than for disyllabic W2s. Although previous studies reported a similar length effect, no satisfactory account of its source has been offered. Some researchers observed that disyllabic Sino-Korean words suppress compound tensification (e.g., C.-M. Lee, 1972), but the length effect extends beyond Sino-Korean words. There are three alternative explanations. First, it is possible that the relatively high frequency of short words (cf. Zipf, 1949) plays a role. Second, we cannot rule out the possibility that Seoul Korean speakers place an AP boundary within long compounds (see Section 1. 2) in some contexts, for example, when one constituent is narrowly focused (cf. Jeon & Nolan, 2017; S.-A. Jun, 1996). The effect of prosodic grouping deserves further investigation, but it was irrelevant to the present study. The participants produced each trisyllabic or tetrasyllabic compound as an AP and there seemed to be no reason for them to divide a tetrasyllabic compound into short APs. In Seoul Korean, trisyllabic or tetrasyllabic APs are common (S.-A. Jun, 1996, 1998, 2005), while an AP is not likely to have seven or more syllables (S. Kim, 2004). Third, a compound’s decomposability is a potential source of the length effect (cf. Kuperman et al., 2010). Longer compounds may bias speakers to decompose them into words rather than treating them as lexicalized words (Laudanna & Burani, 1995), and the decomposition could in turn lead to “loosening” the boundary, hence a decrease in tensification probability (cf. Hay, 2001, 2003; Kuperman et al., 2009, 2010; Lehtonen & Laine, 2003; Libben, 2007; MacGregor & Shtyrov, 2013).

Third, the hypothesis regarding the effect of W2 onset type was partially supported in that tensification was more likely for velar than for bilabial or alveolar W2 onsets, but the W2 onset type effect interacted with the etymology and length effects. The velar effect can be ascribed to the fact that /k/ is the most frequent syllable onset in Korean (see Section 2.3). However, usage frequency may not be the sole reason, since the onsets of the W2-initial syllable /p, tɕ, k/ differ in their articulatory mechanisms. Tensification may occur most effectively when the consonantal constriction is formed in the back of the oral cavity. The velar constriction is formed near the tightly closed vocal folds, and consequently, air molecules can be effectively compressed in between, and this can lead to an explosive release and high burst energy. On the other hand, bilabial consonants are articulated with a longer tube in the vocal tract; therefore, tense articulation may be more difficult because of the rarefaction of the air in the tube.

The source of the interaction between the W2 onset type, etymology and length seemed to be that the trends associated with bilabial and alveolar W2 onsets differed between native and loanword W1s. For native W1s, the probability of tensification was not markedly different between bilabial and alveolar W2 onsets, whereas for loan W1s, the expected ordering of velar /k/ > alveolar /tɕ/ > bilabial /p/ was observed (Figure 4). The reason for such difference is not clear, but it may be the case that either the articulatory restrictions mentioned above for /p/ or its low frequency associated with low tensification probability is further suppressed by other factors, such as the W1’s etymology and long compound length. The probability distributions (Figure 4) for bilabial and alveolar W2 onsets exhibited greater variance for native W1s than for loan W1s. In addition, the variability in tensification probability was particularly low in the two extreme experimental conditions, when the combination of the experimental factors was either most or least likely to lead to tensification. All in all, the variability in compound tensification should not be linearly modeled based on factors, such as etymological class or word frequency; the predictors seem to affect not just tensification probability but also its variability.

Fourth, the presence of a “laryngellay marked” aspirated or tense consonant was associated with a significant decrease in tensification probability. However, it did not interact with W1 etymology (see Section 2.1) and it had only a negligible effect on the W2 onset constriction duration. Ito (2014) analyses this effect as a co-occurrence restriction or obligatory contour principle that close occurrences of segments with similar features, which is (non-modal voicing) for Korean compounds, is avoided (Leben, 1973). This effect seems to be limited to compounds in that in simplex words, co-occurrence of aspirated consonants, but not tense consonants, is restricted (Ito, 2014). However, it may be the case that not just one particular feature, but the constellation of properties, such as longer duration, non-modal voicing and high F0 associated with aspirated or tense consonants make a word with them salient, leading speakers to suppress the emergence of another salient consonant in compounding process (see Payne, 2005 for discussion on how prominence affect realizations of geminates in Italian). At present, the suggestions here are only speculative, and it is not clear which acoustic or articulatory properties cause the suppression effect.

Finally, increases in W1 frequency, W2 frequency and compound plausibility were each associated with an increase in tensification probability. In particular, when a word’s plausibility increased, speakers lengthened the constriction duration of the tensified W2 onset, probably reflecting a further increase in “tightness” of the boundary. This effect was shown only for tense realizations with long duration, probably because they have more scope for temporal adjustment compared to lenis realizations. The implication is that the association between compound tensification involving lengthening in consonantal durations and the tight boundary in speakers’ knowledge may be mirrored in their speech production (cf. Bell et al., 2020; Kuperman et al., 2007; Schuppler et al., 2012).

### 4.2 Compound tensification in contemporary Seoul Korean

Although the origin of compound tensification cannot be empirically tested, understanding its historical development can offer insights into the cause of its variability and the emergence of patterns in the lexicon. Based on an analysis of transcripts, K.-M. Lee and Ramsey (2011) argue that the tense stops were not phonemically distinct in Late Middle Korean in the 15th century (K.-M. Lee and Ramsey, 2011, Section 5.2.1.2), but the lenis consonant at the morpheme boundary was reinforced in various contexts as in present day Korean. In the Early Modern Korean period in the 17^th^ century, the reinforcement was widespread, but it was transcribed for indicating emphasis in meaning (K.-M. Lee and Ramsey, 2011, Section 6.3.1.3; see Sohn, 1999, Section 5.3 for sound symbolism of consonants). It is not clear how the phonologically distinctive three-way distinction emerged, but K.-M. Lee and Ramsey (2011) suggest that native Korean speakers had long been aware of the presence of non-phonemically distinctive tensified sounds. Although tensification did not, and does not change the lexical meaning, its occurrence has been systematic in signaling a morpheme or word boundary in compounds that are not completely lexicalized. The functional load of tensification would have made it transmittable across speakers and learnable. In language acquisition process, learners were exposed to variable inputs, and statistical learning (cf. Pierrehumbert, 2001, 2006) seems to have played a role for the learners to interpret the patterns in data and generalize them over time. At present, compound tensification seems to have been structured as mental representations of phonological targets, as contemporary Korean speakers treat the lenis and tense consonants to be phonologically distinctive.

Taking the findings across studies together, the constituent boundary strength that speakers compute seems to be variable, and that tensification is likely to occur when the constituents form a tight unit. This conclusion is based on the fact that the effects of the constituents’ frequencies (S. Kim, 2017; Zuraw, 2011), plausibility, and familiarity (Ito, 2014) point in the same direction.

Frequent words would have more robust representations in individuals’ lexicon than less frequent words (cf. Bybee, 2001; Pierrehumbert, 2001, 2006), and compound tensification seems to be reinforced in high-frequency items. The effects of W1 frequency and W2 frequency can be interpreted as lexical access of the constituents being used to activate the whole compound (e.g., Juhasz et al., 2003; Zwitserlood, 1994). Although the frequency information of the whole target compounds was not available in the present study, it can be inferred that the high-frequency compounds would be more likely to exhibit tensification. W1 frequency was positively correlated with the number of search results on Google for both W1s and whole compounds. However, the possibility for frequency of the compound and its constituents having independent effects needs to be tested in future studies.

The plausibility effect predicts that when speakers create novel compounds, more plausible compounds are more likely to undergo tensification than less plausible compounds. Plausibility was positively correlated with W1 frequency from H. Kim’s (2005) corpus and also with the whole compound frequencies estimated by the number of Google search results. This suggests that raters’ plausibility judgments may have been influenced by the word’s usage frequency, but plausibility is also applicable to novel words with zero frequency.

The inherently variable and intertwined nature of the tensification triggers would have made previous studies inconclusive. For instance, in Korean, frequent words tend to be shorter than infrequent ones (H. Kim, 2005) as it is the case cross-linguistically (cf. Zipf, 1949). A word’s etymological class is confounded with word frequency in that native words are more frequent than Sino-Korean or loanwords in daily usage. Etymological class is also confounded with the word’s segmental composition; loanwords tend to have more aspirated consonants, which may suppress compound tensification (Ito, 2014) compared to native or Sino-Korean words. Furthermore, native compounds are expected to be rated as more familiar and more lexicalized than compounds in other etymological classes. Native compounds are more likely to be written with *sai-sios* (“between-ㅅ,” e.g., 나루 *nalwu* /nɑlu/ “dock” + 배 *pay* /pɛ/ “boat” as 나룻배 *nalwuspay* /nɑlup*ɛ/ “ferryboat” not 나루배 *nalwupay, see Section 1.2*) following the standard conventions (NIKL, 2017). The written *sai-sios* could prime speakers to produce tensification (cf. Cook, 1987) in native words leading to relatively higher tensification probability. In this regard, the etymological class effect is an amalgamation of a range of partially independent effects. In particular, scale variables are intrinsically inconstant in that word frequency in individual speakers’ lexicon is variable across time, and the perceived familiarity or plausibility will be affected by their experience. Even the choice of the corpus could naturally affect research findings, and inconsistencies in the weighting of various factors across studies are expected. For instance, although the present study showed the interaction between W2 onset type, compound length, and etymology and independent effects of the laryngeally marked consonants, frequency and plausibility, the interaction between factors will be variable depending on the choice of target compounds and study participants.

There are examples in other languages showing that the probabilistic approach has been more successful in predicting the morphological or phonological form (e.g., Kunter & Plag, 2016; Kuperman et al., 2007; Pavlík, 2016; Schuppler et al., 2012) than a set of categorical rules (reviewed in Section 1.2, cf. Chomsky & Halle, 1968) in examining the linking elements in compounds. For instance, there is considerable variation in the choice of Dutch linking morphemes -s- and -en- in noun-noun compounds. Native Dutch speakers’ choice in novel compounds can be predicted based on the distributional probabilities in existing words, primarily on the left constituent (Krott et al., 2001, 2002, 2004) That is, language users utilize their knowledge of the co-occurrence likelihood between the left constituents and linking morphemes in the existing lexicon in drawing analogies. Similar analogical processes seem to be in operation in Korean. In the *wug* tests in the work of Ito (2014), some trends in the existing compounds were mirrored in novel compounds in that tensification was more likely to occur when the W1-final syllable coda was an obstruent than a sonorant and also when there was no aspirated or tense consonant in a compound than when they were present.

While the trends in compound tensification could be modeled with the probabilistic approach, exceptions to the trends exist. Some target compounds showed little variability in their tensification rates in the present study. Eight target compounds out of 72 showed a zero tensification rate. Seven of these were tetrasyllabic compounds with a loan W1, which were in the experimental conditions suppressing tensification. However, one compound /mjʌltɕʰitɕʌt/ “anchovy marinade” is a counterexample; it is a native trisyllabic compound with a high plausibility rating (*M* *=* *4.55*) and a high usage frequency as one of only 19 target compounds listed in H. Kim’s (2005) corpus. Similarly, in the work of Ito (2014), 35 participants unanimously agreed on their tensification judgment for four compounds out of 1,171, although no further details were discussed. These examples show that some compounds are lexicalized as a whole, accepted as existing compounds by language users (cf. Dressler, 2007) with or without tensification.

The discussion so far demonstrates the complex nature of word boundary strength in compounds leading to the phonetic output, but the scope of the present study was still limited. The experimental factors were only a subset of factors proposed in numerous previous studies and they were deliberately selected to keep the scale of the experiment manageable and to vary the structure of the target compounds. For a thorough investigation, the next step would be to conduct an extensive corpus study aiming to build probabilistic models incorporating a wide range of factors with predictive power (cf. Bell et al., 2020), as compound tensification is generalized across new compounds, and possibly to existing compounds. Ito (2014) did not find significant differences in the metalinguistic judgment of compound tensification dependent on the age of participants in Yanbian, China, who were born between 1930s and 1990s. However, in young Seoul Korean speakers’ speech, tensification is increasing; H.-Y. Lee’s (2009) survey carried out with participants in their 20s–70s showed that young generation of Seoul Korean speakers have stronger preference for the tense to lenis pronunciation than older speakers in various contexts, such as word-initially, in a compound, and following /l/ in Sino-Korean words, while between-speaker and-item variation was observed. The variability in tensification does not seem to be restricted to compounds. Such tendency may interact with the spreading of compound tensification.

Modeling the probability and phonetic realization of compound tensification incorporating various factors would allow us to make predictions on its general occurrence across speakers and compounds. However, the relationship between the factors and tensification is not expected to be linear and static. In the course of historical development, compound tensification seems to have been structuralized to some extent, influenced by inherently inconstant factors, such as word frequency. Furthermore, there are some compounds which have been lexicalized with or without tensification, and speakers’ phonetic adjustment may be affected by scale factors, as shown by high plausibility associated with not only higher tensification probability, but also lengthening in the duration of tensified consonants in the present study.

## 5 Conclusion

Much research has been carried out to identify the factors contributing to tensification of a lenis onset consonant occurring at the word boundary in noun–noun compounds (W1 + W2). The vast majority of previous studies relied on authors’ intuitions, and the compound tensification was deemed not entirely explicable or predictable. As the first step of experimental investigation, the present study shows that tensification is a phonological correlate of what speakers judge as a “tight boundary” which is abstract and not physically adjustable by varying speaking rates. However, the “tightness” can further increase duration of a tensified consonant, showing that listeners’ boundary strength calibration has a direct effect on the phonetic form. As demonstrated in existing literature, when tensification occurs seemed to be predictable to some extent, but the effects of scale factors, such as words’ frequency and plausibility indicate that the weightings and interactions between the factors are unlikely to remain constant, and the boundary strength is fluid. There were also lexicalized compounds with or without tensification which were exceptions to the general trends. All in all, compound tensification in Korean shows the complexity of word formation and the relationship between morpho-phonology and phonetic outputs. Further studies using large corpora, and psycholinguistic or neurolinguistics methods will be useful in elucidating the detailed process of compound formation, lexical retrieval, and speech production.

## Supplemental Material

sj-docx-1-las-10.1177_00238309221095479 – Supplemental material for Exploring Variability in Compound Tensification in Seoul Korean Click here for additional data file. Supplemental material, sj-docx-1-las-10.1177_00238309221095479 for Exploring Variability in Compound Tensification in Seoul Korean by Hae-Sung Jeon in Language and Speech

## Appendix

The data for this study are available at: https://uclandata.uclan.ac.uk/329/.

**Table A1.** Target Compounds in Phonemic Transcription with Their Meaning.

W2 onset	Etymology			Tensification (%)	Plausibility
		Monosyllabic W2			
Velar	Native	/noli-kɑm/	“play material”	94.74	4.38
	Loan	/pʰɯlo-kɑm/	“professional material”	56.38	2.06
	Native	/tɑmpɛ-kɑp/	“cigarette price”	96.67	3.98
	Loan	/tʰɛksi-kɑp/	“taxi fare”	96.74	2.62
	Native	/pɑtɕʰu-kuk/	“ cabbage soup”	58.95	4.55
	Loan	/hʌpɯ-kuk/	“ herb soup”	71.88	2.09
	Native	/mʌli-kɯl/	“introduction (lit. head text)”	70.97	4.47
	Loan	/lokɯ-kɯl/	“log text”	33.33	1.91
	Native	/pɑtɑ-kil/	“sea route”	77.42	4.47
	Loan	/kʰʌpɯ-kil/	“curved road”	90.22	4.38
	Native	/pɑtɕi-kit/	“ trouser cuffs”	85.23	3.23
	Loan	/kʰotʰɯ-kit/	“coat collars”	11.70	4.29
Bilabial	Native	/tɕinɛ-pɑl/	“centipede’s feet”	21.11	3.89
	Loan	/tɕʰitʰɑ-pɑl/	“cheetah’s feet”	4.76	2.37
	Native	/susu-pɑp/	“broom-corn rice”	20	4.52
	Loan	/kʰɑlɛ-pɑp/	“curry rice”	18.18	4.56
	Native	/molɛ-pɑt/	“sand field”	4.35	4.55
	Loan	/tɕʰɛli-pɑt/	“cherry field”	8.99	3.65
	Native	/nɑksi-pɛ/	“fishing boat”	36.26	4.55
	Loan	/tɕɛlli-pɛ/	“jelly boat”	13.19	2.01
	Native	/moki-pul/	“mosquito repellent (lit. mosquito light)”	56.63	3.52
	Loan	/kɑsɯ-pul/	“gas light”	6.32	4.55
	Native	/polɑ-pit/	“purple colour (lit. purple light/shade)”	93.75	4.38
	Loan	/hitʰʌ-pit/	“heater light”	21.88	1.93
Alveolar	Native	/nolu-tɕɑm/	“light sleep (lit. roe-deer sleep)”	24.42	2.93
	Loan	/pipʌ-tɕɑm/	“beaver sleep”	6.38	1.41
	Native	/mjʌltɕʰi-tɕʌt/	“anchovy marinade”	0	4.55
	Loan	/mɑŋko-tɕʌt/	“mango marinade”	1.06	1.42
	Native	/sɛlo-tɕul/	“vertical line”	26.37	4.4
	Loan	/lopʰɯ-tɕul/	“rope (lit. rope-string)”	15.38	2.4
	Native	/tɛpʰɛ-tɕil/	“plane-use (lit. plane-act)”	8.51	4.38
	Loan	/tʰɛkɯ-tɕil/	“tagging (lit. tag-act)”	17.71	3.13
	Native	/koki-tɕip/	“meat restaurant (lit. meat house)”	87.1	4.55
	Loan	/pʰitɕɑ-tɕip/	“pizza restaurant (lit. pizza house)”	78.95	4.38
	Native	/kokɛ-tɕit/	“nodding (lit. head-act)”	86.67	4.2
	Loan	/kʰɑpʰi-tɕit/	“copying (lit. copy-act)”	20.45	1.6
		Disyllabic W2			
Velar	Native	/poli-kɑlu/	“barley grounds”	60.47	4.03
	Loan	/kʰʌpʰi-kɑlu/	“coffee grounds”	63.04	3.96
	Native	/nɑmu-kɑtɕi/	“tree branch”	74.47	4.55
	Loan	/okʰɯ-kɑtɕi/	“ oak branch”	0	1.97
	Native	/noli-kʌli/	“play material”	62.64	4.38
	Loan	/njusɯ-kʌli/	“ news material”	73.40	4.47
	Native	/tɕikɛ-kʌli/	“carrier hanger”	9.78	2.23
	Loan	/sɯkʰi-kʌli/	“ski hanger”	0	2.51
	Native	/k*otɕʰi-kui/	“grilled skewers (lit. skewer-grilled)”	2.15	4.55
	Loan	/tɕʰitɕɯ-kui/	“grilled cheese (lit. cheese-grilled)”	9.89	4.12
	Native	/tɕokɛ-kɯlɯt/	“clam plate”	40	2.31
	Loan	/supʰɯ-kɯlɯt/	“soup bowl”	9.78	3.44
Bilabial	Native	/mɑlu-pɑtɑk/	“wide floor”	74.19	4.47
	Loan	/kʰɑpʰɛ-pɑtɑk/	“café floor”	7.45	3.01
	Native	/sulɛ-pɑkʰwi/	“wagon’s wheel”	10.64	4.55
	Loan	/motʰʌ-pɑkʰwi/	“motor wheel”	0	2.67
	Native	/nɛmpi-pɑttɕʰim/	“saucepan trivet”	1.09	4.55
	Loan	/lɛntɕɯ-pɑttɕʰim/	“lens holder”	0	2.93
	Native	/kwili-pʌmpʌk/	“oat mix”	6.38	2.92
	Loan	/pʌtʰʌ-pʌmpʌk/	“butter mix”	0	3.5
	Native	/kɑsi-pɛkɛ/	“thorn pillow”	16.28	2
	Loan	/kusɯ-pɛkɛ/	“goose (feather) pillow”	0	3.62
	Native	/p*ɑllɛ-pinu/	“laundry soap”	59.34	4.55
	Loan	/soltʰɯ-pinu/	“salt soap”	0	3.06
Alveolar	Native	/pɑkʰwi-tɕɑkuk/	“wheel trace”	43.53	4.3
	Loan	/hɛmʌ-tɕɑkuk/	“hammer trace”	46.81	3.01
	Native	/nɑlkɛ-tɕɑli/	“wing spot”	7.61	2.62
	Loan	/tʰɛntʰɯ-tɕɑli/	“tent spot”	7.45	3.54
	Native	/kʌllɛ-tɕokɑk/	“mop piece”	32.98	3.62
	Loan	/pɑksɯ-tɕokɑk/	“box piece”	7.53	2.59
	Native	/kuksu-tɕukʌk/	“noodle spatula”	1.1	2.14
	Loan	/iŋkʰɯ-tɕukʌk/	“ink spatula”	0	2.26
	Native	/tɕʰimɑ-tɕulɯm/	“skirt gathering”	22.11	4.3
	Loan	/sjʌtɕʰɯ-tɕulɯm/	“shirt wrinkles”	2.11	3.86
	Native	/p*ɑllɛ-tɕipkɛ/	“laundry peg”	18.28	4.55
	Loan	/pɛltʰɯ-tɕipkɛ/	“belt tong”	1.08	2.29

The tensification frequency for each compound (%) and plausibility rating means (raw scores) are provided.

## Notes

1. The phonemic transcription in the present paper follows Shin et al. (2013).

2. If the second constituent begins with /i/ or /j/, then /n/ is inserted, for example, /k*ɛ/ “perilla” + /ip/ “leaf” → /k*ɛnnip/ “a perilla leaf.” See J. Jun (2018), Kim-Renaud (1974), and I. Lee and Ramsey (2000, p. 79), among others.

3. In South Korea, the spelling convention regarding compound tensification is complicated as discussed below, whereas it is simply not marked in Yanbian or North Korea.

4. Korean words are Romanized following the Yale conventions.

5. The constriction duration was chosen because it could be reliably measured for both lenis and tense realizations as a potential correlate of the boundary strength, unlike other phonetic parameters discussed in Section 1.1. The relationship between the VOT and the boundary strength would not be straightforward, confounded with constriction duration and voicing; the VOT would be negative for phonetically voiced lenis stops (which are likely to have the shortest closure), a low positive value for tense stops (likely to have the longest closure), and a high positive value for phonetically voiceless lenis stops. For F0, each target compound in the present study was produced as an AP, and therefore, its F0 contour is determined as the AP- and utterance-level intonation (Jeon, 2015 for a review). For voice quality in the vowel following a lenis or tense consonant, it is unlikely that the factors investigated in this study would lead speakers to make very fine adjustments in the glottal configuration and tension (see Gordon & Ladefoged, 2001; Klatt & Klatt, 1990) in a continuous manner.

6. Ito (2014) reported that trends observed in Yanbian which does not transcribe *sai-sios* in writing are similar to those in Seoul Korean. However, the influence of written *sai-sios* on speech production has not been thoroughly investigated.
