# Supplementary material for: Exploring Variability in Compound Tensification in Seoul Korean
Source: Lang Speech. 2022 Jun 3;66(1):214–45. doi: 10.1177/00238309221095479 (PMC9975896; doi:10.1177/00238309221095479)
Supplement: sj-docx-1-las-10.1177_00238309221095479 – Supplemental material for Exploring Variability in Compound Tensification in Seoul Korean [file sj-docx-1-las-10.1177_00238309221095479.docx]

**Supplementary Materials**

*Word Frequency and Plausibilty*

In the online survey about plausibility, the compounds were listed in random order and the participants were asked to rate on a five-point scale whether each compound was likely to be used by the respondent or others. The data were collected anonymously. Cronbach’s 𝛼 was high at 0.94, showing that the participants generally agreed on their ratings (Cronbach, 1951, calculated using the *psy* package, Falissard, 2012, and R ver. 3.6.1, The R Foundation for Statistical Computing, 2019).

The frequencies of W1s (*mean = 3.47, sd = 1.91*) and W2s (*mean = 4.37, sd = 2.16*) showed a positive correlation with the respective Google counts (*W1s, r_s_ = 0.57, p < 0.01; W2s, r_s_ = 0.49, p < 0.01*). W1 Frequency also showed a positive correlation with the compound counts on Google (*r_s_ = 0.40, p < 0.01*). Plausibility Rating (*mean = 1.19, sd = 0.33*) showed a positive correlation with W1 Frequency (*r_s_ = 0.35, p < 0.01*) and the compound counts on Google (*r_s_ = 0.83, p < 0.01*).

Table S1. *Spearman Rank-order Correlation coefficients between the log-transformed frequency measures (G stands for Google) and Plausibility Rating (two-tailed), p ** < 0.01, p * < 0.05, n = 72 for each correlation*

|  | W2 Frequency | G W1 Count | G W2 Count | G Comp Count | Plausibility |
| --- | --- | --- | --- | --- | --- |
| W1 Frequency | 0.02 | 0.57** | 0.05 | 0.40** | 0.35** |
| W2 Frequency | 1.00 | 0.03 | 0.49** | 0.23 | 0.07 |
| G W1 Count |  | 1.00 | 0.22 | 0.38** | 0.14 |
| G W2 Count |  |  | 1.00 | 0.30** | 0.11 |
| G Comp Count |  |  |  | 1.00 | 0.83** |

Table S2. *Summary of the final model with fixed factors, W1 Etymology, W2 Onset Type, and W2 Length, Presence of a Laryngeally Marked Consonant, and participants and items as random intercepts for tensification as a categorical dependent variable, Reference level = W1 Etymology–Native, W2 Onset Type–Bilabial, W2 Length–Monosyllabic, and Laryageal–Not Marked (n = 6,619)*.

|  | Estimate | SE | z value | Pr(>\|z\|) |
| --- | --- | --- | --- | --- |
| Intercept | -7.05 | 1.43 | -4.93 | < 0.001*** |
| Etym–Loan | -0.13 | 1.06 | -0.13 | 0.9 |
| W2 Onset–Alveolar | 0.35 | 1.00 | 0.35 | 0.73 |
| W2 Onset–Velar | 1.98 | 1.02 | 1.94 | 0.05 |
| Length–Di | 0.24 | 1.01 | 0.24 | 0.81 |
| Laryngeal–Marked | -1.07 | 0.53 | -2.04 | 0.04* |
| W1 Frequency | 0.36 | 0.14 | 2.61 | 0.009** |
| W2 Frequency | 0.36 | 0.11 | 3.31 | < 0.001*** |
| Plausibility | 2.26 | 0.83 | 2.72 | 0.007** |
| Etym–Loan × W2 Onset–Alveolar | 1.70 | 1.39 | 1.22 | 0.22 |
| Etym–Loan × W2 Onset–Velar | 1.26 | 1.32 | 0.95 | 0.34 |
| Etym–Loan × Length–Di | -4.01 | 1.61 | -2.49 | 0.01* |
| W2 Onset–Alveolar × Length–Di | -0.98 | 1.40 | -0.70 | 0.48 |
| W2 Onset–Velar × Length–Di | -1.11 | 1.30 | -0.85 | 0.39 |
| Etym–Loan × W2 Onset–Alveolar × Length–Di | 2.38 | 2.12 | 1.12 | 0.26 |
| Etym–Loan × W2 Onset–Velar × Length–Di | 1.91 | 2.03 | 0.94 | 0.35 |

Table S3. *Results of the log-likelihood tests with fixed factors, W1 Etymology, W2 Onset Type, and W2 Length, Speaking Rate, Presence of a Laryngeally Marked Consonant, Plausibility Rating, W1 Frequency and W2 Frequency for constriction duration (ms) as a dependent variable (lenis, n = 4510; tense, n = 2108)*

|  |  | lenis |  | tense |  |
| --- | --- | --- | --- | --- | --- |
|  | df | χ^2^ | Pr(>χ^2^) | χ^2^ | Pr(>χ^2^) |
| Etym × W2 Onset × Length | 2 | 0.27 | 0.87 | 2.29 | 0.32 |
| Etym × W2 Onset | 2 | 0.08 | 0.96 | 1.31 | 0.52 |
| Etym × Length | 1 | 1.82 | 0.18 | 1.36 | 0.24 |
| W2 Onset × Length | 1 | 0.15 | 0.93 | 4.77 | 0.09 |
| Etym × Laryngeal | 1 | 4.28 | 0.04* | 2.75 | 0.10 |
| Etym | 1 |  |  | 0.07 | 0.79 |
| W2 Onset | 2 | 82.21 | < 0.001*** | 37.44 | < 0.001*** |
| Length | 1 | 25.58 | < 0.001*** | 50.54 | < 0.001*** |
| Speaking Rate | 1 | 185.25 | < 0.001*** | 496.12 | < 0.001*** |
| Laryngeal | 1 |  |  | 1.40 | 0.24 |
| W1 Frequency | 1 | 1.35 | 0.24 | 1.09 | 0.30 |
| W2 Frequency | 1 | 0.36 | 0.55 | 0.02 | 0.90 |
| Plausibility | 1 | 3.62 | 0.06 | 5.76 | 0.02* |
